# Treatment of the Insane*Read at the Waco Meeting of the Central Texas Medical Association, July, 1900.

**Published:** 1900-10

**Authors:** Frank R. Ross

**Affiliations:** Assistant Physician State Insane Asylum, Austin, Texas


For Texas Medical Journal.

### Treatment of the Insane.*

BY FRANK R. ROSS, M. D., ASSISTANT PHYSICIAN STATE INSANE ASYLUM, AUSTIN, TEXAS.

It was not until almost the close of the eighteenth century that insanity was given the dignity of a disease. While it is true that insanity was regarded by many early writers as a disease, its status as such was not generally accepted until after the notable reform of Pinel in 1793.

The treatment of the insane has been varied, and a brief reference to the customs existing in earlier days I trust will not be amiss.

Looking backward through the dim vista of past ages, long before the birth of Christ, we find that these unfortunates are kindly treated. Referring everything to celestial intervention, it was believed that the insane were possessed of a benevolent or an avenging deity, and their treatment was accordingly given over to the priests. In Greece the Asclepiades, or medical priests, practiced their religious ceremonies by beseeching the investing deity to depart, and subjecting the patient to expiations, ablutions with the lustral water, or the blood of the sacrificial victim. Occasionally there was coupled with this practice certain hygienic principles, such as exercise in the gymnasium, music, spectacles and bathing in hot springs. If it so happened that a cure was effected, it was consid-

*Read at the Waco Meeting of the Central Texas Medical Association, July, 1900.

ered that the deity was appeased, and rich offerings were made, which, for the most part, were appropriated by the priests.

Similar beliefs and practices prevailed with the Egyptians. Subsequently these notions were handed down and retained by the priesthood, and there was little variation in the practice until Hippocrates, himself a member of the Asclepiades, came upon the scene.

He may be said to be the originator of mental medicine, and it was he who first gave in a crude way classification to the insanities. He describes idiopathic and symptomatic insanities, acute febrile delirium, mania, melancholia, alcoholic and the insanity of pregnancy.

While his writings lack clearness, especially as regards mania and melancholia, it is evident he recognized these forms. He possessed some faint notions of hysteria, and deals clearly with epilepsy.

Recognizing the pathological nature of insanity, he persistently opposed the ideas of the Asclepiades until his theories prevailed, and the ablutions and incantations were replaced by phlebotomy, emetics, purgatives, baths, diet, music and various diversions,—such were the therapeutic measures of the day.

Certain drugs were supposed to be specifics for insanity, for instance, hellebore (veratum album), was collected by his patients at Anticyra, a village in Thessaly, where could be found the most reputed variety. Mondragora, afterwards famed in witchcraft, was the special drug used in cases of suicidal melancholia, it being regarded as specific.

Nothing is known of the management and care of the insane at this time, but it is supposed that restraint in its extreme form was practiced, and there was no place for their detention.

The doctrines and beliefs of Hippocrates prevailed for several hundred years afterwards, and there was no improvement or advancement in this line, as those who followed did nothing more than imitate his methods. It was likewise in Egypt. Some several centuries later the Romans reached the highest pinnacle in the scientific investigation of mental disease, and their writers from several years before the birth of Christ, and for several centuries afterwards, described with notable accuracy and minuteness insanity in many of its phases.

In management and treatment the highest perfection is noted, and in this respect they approached in excellence and scientific understanding the methods in use today. Strict non-restraint was advocated and practiced, hygienic and moral measures were extolled; gentleness and kindly usage were considered more effective than bodily violence and blows. Such treatment as the latter, it was argued, would not return the reason, but would "only aggravate the patients' condition, injure them physically and offer to them the miserable remembrance of their sufferings whenever they recovered the use of their reason." Whenever it became necessary to use restraint, as was sometimes required with disturbed and violent patients, every precaution was taken to prevent injury; the joints were covered with some soft substance and the patient was tied.

Gradually the study of insanity declined, until after the tenth century everything relating to it seemed to fall into obscurity and oblivion.

Witchcraft, demonopathy and lycanthropy came to be the beliefs of men, and superstition ran riot with reason. Woe to the unfortunate whose mind sickened!

Most revolting and terrible was the treatment of thousands who lost their lives during this gloomy and shadowy period. We see the ill-fated lunatic bound with chains, thrown into some dark dungeon with its foul and mephitic air, a bundle of rotten straw for a bed, confined and treated like some wild beast, an object of curiosity and detestation, until, if by this treatment the demon was not exorcised, the sufferer was bound to the stake and burned, or executed in some other way. Not a protest was heard. A religious frenzy possessed the minds of men, and with ignorance and prejudice dominated their every action. Parliaments were even more blood-thirsty than the populace, and many laws were passed perpetuating these barbarious customs.

But slowly a reaction set in from these extremes, but it was not until the fifteenth century that a more healthy turn of affairs began.

Timidly at first, but bolder and more courageous as time wore on, the medical men urged amelioration in the treatment of the insane, and they began again to study mental medicine, but very little was accomplished. The people continued to throw the insane into dungeons, and to torture and execute them.

A brighter day was to dawn, however, for this much abused and ill-fated class, but it was not until 1793, Phillip Pinel and his co-worker, Pussin, vigorously protested against the manner of treating the insane in France. Pleading earnestly for the separation of this class from criminals, and their confinement in institutions, and after clearly demonstrating the necessity of this glorious innovation, his efforts were successful, and a new era in the treatment of the insane was begun.

This reform was not confined to France alone, for almost simultaneously a like movement was begun in Italy by Chiarruggi, and in England Wm. Tuke, in conjunction with the Quakers, founded an institution for the care of the insane, from which all severe restraint was excluded. In America, Benjamin Rush was at this time striving for the better treatment of these people. Unfortunately, the humanitarian principles promulgated by these men were slow to be adopted. Prejudice and superstition, with the belief in witchcraft and demons, were too tenaciously rooted in the minds of the populace to soon give place to the broader, higher and nobler teachings of these gentle reformers.

Though the seeds of humanity were sown on crude and rough soil, they were destined to take root, and in time reach a bountiful fruition.

For the next few decades after Pinel's reform was launched, in England, America and elsewhere the insane were not so persecuted, but their chains were not all removed, for as late as 1843 there was found in one French asylum fifteen men and twenty women naked and chained in their cells. Still, a better turn of affairs was established, and everywhere institutions for the care and treatment of the insane began to be erected. States are providing liberally in the way of buildings, thorough equipment and ample maintenance funds.

Distributed everywhere are asylums, county and State, poor houses with a department for this class, private asylums and sanitariums, and various other places of nondescript character where patients are received and treated.

With the beginning of this segregation of the insane, the study of mental medicine was pursued with renewed energy, and the last few years have been prolific in new ideas, and there has been a general enlightenment on this subject. Much investigation in the etiology and pathology has been carried on, and the disease is now better understood, and as a result marked changes have taken place in all institutions, both as regards treatment and control.

In many until a few years ago, and even now in some places, depressants, hypnotics and mechanical restraint were the chief therapeutic measures in use. Such remedies are decidedly detrimental to any case of insanity.

Restraint by mechanical means is particularly damaging. In disturbed and violent cases, there is usually a condition of hyperemia of the brain which disturbs the normal metabolism, resulting in trophic changes in the higher nerve cells.

The toxic and deleterious materials generated surcharge the blood, and when carried to the brain their irritant effects are manifested by excesses of some form; it may be violent muscular movements or incoherent and hightened mental activity.

To mechanically restrain such a case would be like adding "fuel to the flames,"—the excitement is aggravated, the abnormal biochemical changes are increased by the pent-up energy, and all together conditions are rendered decidedly worse.

Again, pour into the stomach of this patient depressants and hypnotics (hyoscyamus, hyoscine or hyoscyamine, cannabis indica and the bromides) and see the result. Disturbed digestion and absorption of the toxines generated thereby, anorexia, sleeplessness, all tending to increase morbid cell changes, until exhaustion supervenes and death closes the scene. If not this, a chronic condition, or dementia (a mental death) ensues, due to the irreparable injury to the brain and nerves. This picture is doubtless strongly drawn, but this practice has existed in years gone by, and hand-cuffs, restraint chairs and beds and other mechanical means of restraint, with depressants and hypnotic drugs, preponderated as therapeutic measures.

This practice has been almost entirely abandoned. The treatment of today being strict non-restraint, tonics, generous feeding, hydrotherapy, diversions, exercise and occupation. Now, when restraint becomes absolutely necessary patients are controlled by skilled attendants. Control by skilled attendants should be devoid of harm or injury, and the moral effect of this means of control is splendid on the patient. It is seldom that their efforts at self will continue for any length of time, and the most violent soon yield to hospital discipline.

Sleep and rest are essential to repair of tissue, and this repair is better when the sleep is normal, but when produced by artificial means, these processes are more or less hindered; still, it becomes necessary at times to employ hypnotics. They are all more or less depressing. Chloral produces sleep which is like natural sleep, but it is uncertain and dangerous, and is distinctly contra-indicated where organic heart disease exists. The bromides are likewise depressing, and are objectionable because of the tendency to produce digestive disorders.

Hyoscyamus, hyoscine and hyoscyamine are not free from objections. Paraldehyde seems to be a safe sleep begetter, and is only contra-indicated in gastric affections. Its hypnotic effect is somewhat evanescent, and it leaves an unpleasant odor to the breath.

Sulfonal is slow but effective, and is not entirely devoid of ill effects. So with trional, which, though comparatively safe, when long administered produces a toxic substance in the blood, called haematoporphyrin, which is a direct irritant to the kidneys. The latter drug, however, has proven more satisfactory with us than any of the others mentioned.

In every institution there are found patients who are unruly and noisy, and who resist all discipline, and for this class there is no better remedy than the hypodermic use of the hydrochlorate of apormophine in 1/10 grain doses. The medicine acts quickly, usually producing emesis in from five to ten minutes. So profound is the impression produced, and so great the dread inspired, that the dose seldom has to be repeated for several months. Its moral effect is excellent. Patients who could not be controlled by other measures, and who persisted in fighting or otherwise creating disturbances in the wards, are quieted by one administration of the drug. It was first used for this purpose by Dr. B. M. Worsham.

Exercise is very important in the treatment of the insane, and active exercise for irritable and excitable patients furnishes a normal and healthy outlet for surplus energy, stimulates the excretory functions to a better elimination of the waste products, and by reason of the fatigue engendered induces a condition of quietude favorable to rest and sleep.

Exercise, supplemented by generous feeding with nourishing and easily digested food, with tonics, aid in restoring tone to the irritable nerve cells.

Medicinal tonics, iron, strychnine, hypophosphates, gentian, cinchona and others aid greatly in the process by enriching the impoverished blood by their stimulating effects on the digestive functions, and by increasing assimilation. The effects, direct and indirect, derived from medicinal tonics are of immeasurable good in combatting debility, in diminishing irritability and in conserving nerve force. In extreme depressions with a tendency to suicide, as an acute melancholia, it is often necessary to use sedatives in conjunction with the afore-mentioned remedies, and there is no better sedative for this condition than the aqueous extract of opium.

Constipation must be met with purgatives and laxations, as the case may require.

Diversions play a most important part in preventing intro-spection (a condition present in nearly all acute cases), and in keeping their thoughts from dwelling on their condition. Accordingly, it is the custom in institutions to have weekly dances, serenades, picnics and entertainments of various kinds. Pianos and organs are found in the women's department, and billiard tables are provided for the men. Games of all kinds are given to them, and they are urged to divert themselves in some way, all with a view of inducing a healthier trend of thought, to cause forgetfulness of their affliction, and to give pleasure to those whose minds are lost irretrievably.

Occupation has reached an advanced place as a curative measure for the nervous and insane. It, too, serves to distract, and procures relief from depressed feelings, uses up surplus energy in a normal way, and is conducive to rest and sleep. Appetite is promoted, and digestion and assimilation are made better. For destructive cases there is no better preventive to their morbid tendencies than manual labor. Patients who persist in tearing their clothes, disfiguring the walls and destroying everything in reach are greatly benefitted by work. All of these vicious traits and imperative ideas are allayed and quieted, and the patient takes on better habits.

It is useless to say that work is not indicated in exhausted conditions, nor should one be allowed to go to this extreme. Work is not compulsory in institutions, but the patients are urged to engage in some occupation. Occupation therapy is undoubtedly rational, and it is destined to be practiced more extensively, not only with the insane, but in a great many nervous disorders, especially in hysteria and neurasthenia. The question how to best care for the large class of chronic and dependent insane is yet to be solved.

Their custodial care is a great expense upon the people, and anything that will lessen this burden and at the same time give some degree of usefulness to these useless lives is an advancement of the greatest importance.

To make them producers and self-sustaining is the central idea, and with this in view farm lands have been purchased by some of the southern institutions, and colonies composed of those patients able to work have been established, and they are engaged in raising produce of all kinds. Vegetables, fruit, corn, dairy products, chickens, hogs and beef are raised on the farms and consumed in the institution. This, of course, means a reduction of the per capita cost, and a smaller appropriation for maintenance, and a saving for the people.

This plan has not yet been adopted in this State, but it is expected that the epileptic colony at Abilene will be conducted somewhat on this plan, and its acceptance for the institutions for the insane has been earnestly urged by Dr. B. M. Worsham, and it is confidently expected that his efforts in this direction will be successful, when, at the next session of the Legislature, money will be appropriated for the purchase of the necessary farm lands and the erection of cottages. Then the colony system will be launched, and Texas will rank with the foremost of the States in providing and caring for the dependent insane.

